# Unveiling the future of breast cancer assessment: a critical review on generative adversarial networks in elastography ultrasound

**DOI:** 10.3389/fonc.2023.1282536

**Published:** 2023-12-06

**Authors:** Mohammed Yusuf Ansari, Marwa Qaraqe, Raffaella Righetti, Erchin Serpedin, Khalid Qaraqe

**Affiliations:** ^1^ Electrical and Computer Engineering, Texas A&M University, College Station, TX, United States; ^2^ Electrical and Computer Engineering, Texas A&M University at Qatar, Doha, Qatar; ^3^ College of Science and Engineering, Hamad Bin Khalifa University, Doha, Qatar

**Keywords:** generative adversarial networks, elastography ultrasound, breast cancer diagnosis, enhancing pocket ultrasound, computer-aided diagnosis, artificial intelligence in medical imaging, medical image synthesis, image-to-image translation

## Abstract

Elastography Ultrasound provides elasticity information of the tissues, which is crucial for understanding the density and texture, allowing for the diagnosis of different medical conditions such as fibrosis and cancer. In the current medical imaging scenario, elastograms for B-mode Ultrasound are restricted to well-equipped hospitals, making the modality unavailable for pocket ultrasound. To highlight the recent progress in elastogram synthesis, this article performs a critical review of generative adversarial network (GAN) methodology for elastogram generation from B-mode Ultrasound images. Along with a brief overview of cutting-edge medical image synthesis, the article highlights the contribution of the GAN framework in light of its impact and thoroughly analyzes the results to validate whether the existing challenges have been effectively addressed. Specifically, This article highlights that GANs can successfully generate accurate elastograms for deep-seated breast tumors (without having artifacts) and improve diagnostic effectiveness for pocket US. Furthermore, the results of the GAN framework are thoroughly analyzed by considering the quantitative metrics, visual evaluations, and cancer diagnostic accuracy. Finally, essential unaddressed challenges that lie at the intersection of elastography and GANs are presented, and a few future directions are shared for the elastogram synthesis research.

## Graphical Abstract

Overview of the GAN framework employed by Yao et al. (20) and Yu et al. (40) for accurate breast lesion elastogram synthesis, aiding in accurate diagnosis of detected lesions in US image.

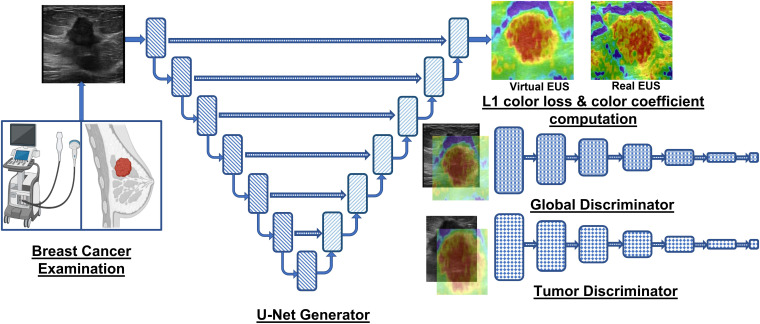

## 1 Introduction

Ultrasound (US) imaging is commonly applied across diverse clinical environments for visualizing various anatomical regions within the human body. US modality operates on the principles of reflection and scattering of highfrequency ultrasound waves from different types of soft tissues (of varying echogenicity) within the human body. US imaging presents numerous advantages that make it a favorable alternative to other medical imaging modalities (e.g., X-ray (1), magnetic resonance imaging (MRI) (2), computed tomography (CT) (3), and histopathology images (4, 5)). These advantages include its cost-effectiveness, patient safety, widespread availability, exceptional diagnostic efficacy, user-friendliness, portability, and, notably, its radiation-free nature (6).

Elastography Ultrasound (EUS) adds additional information regarding tissue elasticity to the conventional gray scale Ultrasound (also known as the B-mode US) (7). In a typical EUS, a tissue compression mechanism is used along with the transducer to assess tissue stiffness or elasticity. The response of tissue to mechanical deformation or vibration is processed and visualized as a color-coded map to quantify tissue stiffness. The type of algorithm used to generate the elasticity color map depends on the elastography technique utilized in EUS. For instance, strain-based elastography (8) (i.e., mechanical deformation) utilizes correlation-based methods, which calculate the displacement or strain by comparing pre-compression and post-compression US images. Shear wave elastography (9) employs time-of-flight methods, measuring the time shear waves (generated by the transducer) take to propagate through the tissue. Acoustic radiation force impulse (10) applies a localized acoustic radiation force to the tissue and measures the resulting tissue displacement using cross-correlation or speckle tracking algorithm. Model-based elastography techniques (11, 12), employ mathematical models to estimate tissue stiffness based on the data acquired from the US images. Subsequently, the strain information in the generated elastogram about the region of interest (ROI) is studied by radiologists to diagnose diseases such as liver fibrosis (13), breast lesions (14, 15), prostate cancer (16), thyroid nodules (17), and musculoskeletal disorders (18, 19). Specifically in the case of breast cancer, the elastogram allows the radiologists to accurately identify stiffer ROI (i.e., malignant lesions), minimizing the removal of benign lesions and damage to healthy tissues in biopsies. Moreover, the lesion shape, infiltration pattern, and elasticity analysis of surrounding tissue may provide important information regarding the extent and aggressiveness of the carcinoma, thereby guiding treatment decisions. Altogether, B-mode ultrasound provides anatomical information, and elastography adds the perspective of tissue stiffness or elasticity, increasing the clinical utility of US.

The integration of elastogram into the US enhances its clinical applicability and utility but introduces several new challenges. B-mode US is subjective to the radiologist’s experience and expertise. The sensitivity to human subjectivity and expertise increases significantly for EUS because of additional factors during US capture, such as probe position, applied pressure, and frequency of mechanical compression (20). Furthermore, radiologists require additional training to accurately interpret the elasticity information and differentiate pathologies (i.e., types of tissue) in the color-coded heatmaps. The elastograms are also influenced by signal attenuations, which degrades the quality of EUS for deep-body tissues. Therefore, radiologists need to be familiar with the artifacts in EUS to provide accurate diagnoses while correlating their findings with the patient’s clinical history.

Deep learning algorithms have revolutionized the analysis of US images because of their automatic nature, ability to extract task-relevant features (i.e., reduced dependence on domain knowledge), state-of-the-art performance, and end-to-end nature (21). However, the well-known neural network-based methodologies face challenges due to the composition and noise in US images, which are typically absent in real-world natural images (22, 23). To elaborate, the typical grainy texture of US images is due to the salt-pepper or speckle noise arising from the interference of reflected sound waves (24). In addition, US images may also contain reverberation artifacts due to the sound waves echoing from two strong anatomical structures, resulting in duplicate structures (25). Furthermore, a bone or calcification can prevent the passage of sound waves, leading to incomplete visualization of the underlying tissues (26). Apart from the noise and artifacts in US images, two different anatomical structures (e.g., pancreas and liver) may appear to be the same depending on the probe position and view of the US, making US analysis challenging for radiologists and deep learning models without probe location metadata.

Recently, Yao et al. (20) have proposed a scheme to generate EUS images (i.e., elastograms) from the conventional B-mode US using a GAN to improve breast cancer diagnosis and the utility of pocket US. In this critical review, we thoroughly examine the work carried out by Yao et al. (20) in the field of EUS image synthesis. The review incorporates various crucial aspects, including a thorough comparison with relevant prior studies in medical image synthesis, a concise overview of the GAN methodology for EUS synthesis, an extensive analysis of the results, and a comprehensive discussion of the unaddressed challenges and potential future directions in EUS generation. By critically evaluating this methodology, our aim is to provide an insightful analysis of the current state-of-the-art in the synthesis of EUS images while also shedding light on the areas that require further investigation and improvement.

The remainder of this critical review is structured as follows: Section II specifies the contributions of Yao et al. (20) and the impact of synthesized EUS. Section III provides an overview of the state-of-the-art medical image synthesis and compares it with the methodology proposed by Yao et al. (20). Section IV describes the GAN methodology, loss functions, and metrics for evaluating the generated V-EUS. Section V presents an analysis of the vital results that support the claims of Yao et al. (20). Section VI discusses un-addressed challenges and essential future directions. Finally, section VII concludes the critical review.

## 2 Contributions and impact

The key contributions of the methodology proposed by Yao et al. (20) are next summarized. First, the manuscript proposes a GAN for synthesizing virtual EUS (V-EUS or synthesized EUS) from B-mode US. Notably, the authors provide an alternative to conventional EUS generation which could improve the clinical impact of portable US (27, 28). Second, the methodology enhances the GAN network with a tumor discriminator module and a color balancing module, allowing the network to differentiate between the tumor and healthy tissue while ensuring the V-EUS possesses a color distribution that aligns with the actual EUS image. Third, the proposed GAN model is meticulously trained and evaluated using an extensive patient cohort from fifteen medical centers. The dataset comprises 4580 cases, with 2001 images utilized for training, 500 images for internal validation, and 1730 cases from 14 centers for external validation. Furthermore, 349 extra cases of pocket US are employed to evaluate the generalizability in pocket US setups. Fourth, the generated V-EUS images undergo comprehensive testing using quantitative metrics (e.g., image similarity) and qualitative analysis (i.e., visual evaluation). The applicability of the V-EUS is also demonstrated in real-world scenarios, such as improving breast cancer diagnosis, generating elastograms for deep tissues, and improving the diagnostic effectiveness of pocket US.

The impactful contributions of Yao et al. (20) advance academic knowledge, influence existing usage and protocols of the US for breast cancer diagnosis, improve the standard of healthcare in society, and inspire new research frontiers. Also, the authors propose an additional tumor discriminator, which takes the tumor area as the input and determines the authenticity of the tumor region. Additionally, the L1 loss between the V-EUS and real EUS is reweighed using a computed color coefficient to account for color rarity in elastograms. These innovations allow the GAN framework to render color-accurate elastogram of tumor and neighboring tissue, which can also be extended to synthesize EUS of tumors in abdominal organs (e.g., hepatocellular carcinoma) with appropriate training data. The successful reconstruction of V-EUS by the GAN framework, despite the prevalent noise and artifacts in breast US images, significantly impacts the existing protocols of the US breast cancer diagnosis. Particularly, the generation of accurate elastograms for deep-seated tumors, where conventional elastography setups yield suboptimal results due to signal attenuation, signifies a breakthrough. Moreover, the integration of the GAN with pocket US devices can make elastography accessible on portable US platforms, which was not possible earlier due to limited hardware and computational power. Subsequently, the availability of V-EUS for pocket US holds profound societal implications as it can improve the diagnostic accuracy of breast cancer in small clinics and mobile mammography units while providing malignancy information of the detected tumors, thereby shrinking the time duration of the diagnostic protocols and allowing for early and effective treatment. Lastly, a noteworthy impact of this research lies in its potential to inspire innovative GAN variants tailored for elastography generation of other anatomical structures to improve the diagnosis of other carcinomas and fibrosis in a prompt, cost-effective, and timely manner.

## 3 Literature comparison

Deep learning models have achieved notable success in classifying, segmenting, and detecting relevant ROI in medical images and other modalities of data (2, 29–33). Recently, neural networks have been employed to upscale low-resolution medical images, transform medical imaging modalities, enhance visualization, and improve diagnostic accuracy. Muckley et al. (34) present key learnings from the 2020 fastMRI challenge, which aimed at accelerating the development of neural network architectures for MR image reconstruction while providing a fair open-access comparison to the research community. The manuscript highlights that error characterization and AI-generated hallucinations are critical challenges in evaluating MR images generated by neural networks. Qu et al. (35) propose the WATNet architecture to generate 7T MRI (i.e., improved anatomical details) from 3T MR images by combining information in spatial and wavelet domains. Notably, the WAT modules learn the scaling and translational parameters for each pixel in the feature map based on the wavelet coefficients, allowing the network to scale different regions of the feature map based on the contrast and edge information in the frequency domain. The WAT module can also serve as a prior for other image synthesis tasks such as CT to MRI conversion. Similarly, Li et al. (36) propose a two-stage deep learning framework, employing 3D-UNet and convolutional LSTM, to accurately reconstruct thin-section MR images from thick-slice MR images, specifically targeting brain MRI super-resolution. High-level methodology analysis reveals that these works employ conventional fully convolutional network (FCN) designs for image reconstruction and superresolution tasks. However, compared to FCN architectures, GAN-based approaches offer several advantages. GANs facilitate sophisticated implicit feature learning within the generator, enabling the network to capture complex patterns from medical images. Moreover, the adversarial training paradigm further enhances the network’s ability to learn and generate realistic and high-fidelity medical images.

Recently, GANs have been employed to add an extra dimension to histopathological images. Rivenson et al. (37) employed GANs to transform wide-field autofluorescence images into their corresponding stained versions. An exhaustive evaluation of the GAN on the salivary gland, thyroid, kidney, liver, and lung, involving different stains, shows that virtual staining can circumvent labor-intensive and costly histological staining procedures without any significant differences from the real stained images. Inspired by this application to enhance histopathology, researchers have employed GANs to generate EUS without requiring conventional US setup. Zhang et al. (38) propose a GAN framework, termed AUE-Net, with a U-Net generator equipped with attention mechanism and residual connections for a compelling depiction of elastograms for thyroid nodules. The spatial attention module is utilized at the beginning of the U-Net to identify the nodule regions, and a color attention module is used at the end to create a color attention map for EUS. Moreover, the loss function of the network is augmented to account for the color difference between the real and generated elastograms, forcing the generator to produce images with a color distribution that overlaps real elastograms. Despite the significant contributions of AUE-Net, Yao et al. (20) present essential improvements to the methodology design, evaluation, and application of GANs for elastogram generation. Specifically, the use of a tumor discriminator enables the network to identify tumor areas with higher precision relative to the spatial attention module, which is reflected in the qualitative analysis of the generated elastograms. Additionally, Yao et al. (20) enhance the color loss by using the lab color space with a mathematically derived color coefficient to account for color rarity. Moreover, the authors evaluate the quality of generated elastograms based on improved breast cancer diagnostic accuracy, elastography of deep-seated tumors, and improvement in diagnostic effectiveness of pocket US, which were omitted in the evaluation of AUE-Net for the elastography of thyroid nodule. In a complementary study, He et al. (39) investigate the suitability of using a GAN-based approach (i.e., SRRFNN) to improve lateral resolution in the radiofrequency (RF) data (i.e., up-sample RF data perpendicular to acoustic beam), consequently improving the elastogram quality in ultrasound strain elastography. However, the V-EUS (20) generation approach is a preferable end-to-end solution because it generates elastograms directly from conventional B-mode US rather than upsampling the lateral resolution to improve quality. As an extension to the contributions of Yao et al. (20), Yu et al. (40) utilize the same GAN framework and dataset to show the feasibility of V-EUS in augmented reality (AR-EUS) for improved diagnosis of breast cancer with pocket US. The quantitative and blind evaluation of elastograms in augmented reality shows no significant discrepancies between the AR-EUS and real EUS, establishing the authencity of AR-EUS. **Table 1** summarizes the state-of-the-art methods in medical image synthesis that laid the pathway for GAN framework proposed by Yao et al. (20).

**Table 1:** Literature overview of the state-of-the-art methods for medical image synthesis.

Reference (Year)	Task	Dataset Information	Core Methodology	Remarks
Rivenson et al. (2019) (37)	Virtual staining	Whole slides of 211,475 and 59,344 of Liver and Kidney tissues	GAN framework, U-Net generator combined with an FCN discriminator	**Pros:** GAN-generated virtual staining can provide similar results as conventional staining, providing time and cost-saving **Cons:** The GAN framework is not validated for other contrast-generating methods multiple excitation and emission wavelengths
Muckley et al. (2020) (34)	MR image reconstruction	7,299 clinical brain scans subsampled k-space data	Comparative analysis of networks for MR image reconstruction for fastMRI challenge 2020	**Pros:** Deep learning methodologies decrease the minimum requirement for MR image reconstruction set by parallel imaging and compressed sensing methods **Cons:** Pseudo-regular sampling of the MR data lacks realism and is not equivalent to the perfectly equidistant sampling pattern used on MRI systems
Qu et al. (2020) (35)	Image enhancement (Image super-resolution)	15 pairs of 3T and 7T brain images	WATNet, an encdoer decoder network with wavelet priors and conditional normalization	**Pros:** Wavelet coefficient can allow learning feature map normalization weights **Cons:** Other tasks, such as MRI to CT and T2 images from T1 translation have not been explored in the work
He et al. (2020) (39)	RF super resolution	50 human subjects, 50-90 frames per patient	Super-resolution radio-frequency neural network (SRRFNN) inspired by a super-resolution GAN	**Pros:** Laterally upsampled RF data processed by SRRFNN performs better than conventional bi-cubic interpolation approach **Cons:** The method does not utilize the actual high-frequency US data using novel beam-forming technology for training
Li et al. (2021) (36)	MR image reconstruction	305 paired brain MRI samples with a thickness of 1.0 mm and 6.5 mm	3D U-Net followed by a convolutional LSTM network for MRI slice refinement	**Pros:** Practical and clinical value of generated thin MRI is higher than other voxel-based morphometry **Cons:** The quality of reconstruction is directly dependent on the accuracy of statistical parametric mapping (SPM)
Dalmaz et al. (2022) (41)	MRI to CT translation, MRI missing slices generation	IXI dataset (53 subjects), BRATS dataset (55 subjects) (42), multi-modalpelvic MRI-CT dataset (15 subjects) (43)	ResViT architecture with vision transformers’ block at the bottleneck and convolution operators in the encoder and decoder of the GAN generator.	**Pros:** Convolutional and transformer branches within a residual bottleneck of the generator preserves both local precision and contextual sensitivity **Cons:** Architecture needs further validation with unpaired sets of medical images using cycle consistency loss.
Ozbey et al. (2022) (44)	MRI to CT translation	IXI dataset (40 subjects), BRATS dataset (55 subjects) (42), multi-modal pelvic MRI-CT dataset (15 subjects) (43)	Adversarial diffusion modeling using conditional diffusion for capturing and correlating the image distributions.	**Pros:** Cycle-consistent architecture is used with coupled diffusive and non-diffusive components to bilaterally translate between imaging modalities. **Cons:** Adversarial loss in diffusion models introduce training instability and suboptimal convergence
Zhang et al. (2022) (38)	Elastogram generation	726 thyroid US elastography images of 397 patients	AUE-Net GAN framework, U-Net generator with spatial and color attention.	**Pros:** L1 loss can be added to the generator loss for improving the color distributions of generated elastograms **Cons:** The method does not perform qualitative evaluation of the generated elastograms
Yu et al. (2023) (40)	Elastogram generation	4580 breast cancer cases from 15 medical centers	GAN with a U-Net generator, global and local tumor discriminator, with L1 loss and color coefficient	**Pros:** AR-EUS improves the diagnosis accuracy of pocket US **Cons:** The GAN framework has been only validated for the Chinese population

## 4 Methodology overview

GANs are a new class of neural network architectures that excel at generating high-fidelity new data (e.g., elastograms from US images). In terms of architecture, GANs differ significantly from the conventional FCNs because they contain two subnetworks, which are trained adversarially to enhance the capability of the system to generate realistic data instances. To elaborate, a brief description of the components of GANs is next presented. **Graphical Abstract** describes the neural network architectures of the generator and discriminator within the GAN framework proposed for elastogram synthesis.

### 4.1 Generator

In EUS synthesis, the generator is an encoderdecoder architecture that generates realistic synthetic elastograms (i.e., V-EUS. Specifically, U-Net architecture (45) is a popular choice for a generator because of its capability to capture multi-scale features and low-level features (through skip connections) to generate elastograms. The encoderdecoder design of the U-Net allows for parameter savings due to shrinking spatial dimensions of the feature maps in the deeper layers of the encoder, thereby providing computational savings. Yao et al. (20) employ the vanilla U-Net architecture with tuned channel count in the encoder and decoder for the generation of elastograms.

### 4.2 Discriminator

The discriminator of the GAN framework is an FCN that receives the output of the generator (i.e., elastogram) or real EUS as input and performs binary classification. Yao et al. (20) employ a sophisticated discriminator paradigm derived from conditional GAN, which adds the B-mode US image as an additional input (i.e., prior knowledge) to the discriminator network, enhancing its ability to differentiate between real or V-EUS. The authors also add a local tumor discriminator to the framework to further enhance the capability of the system to distinguish between real or fake tumor areas and their elastograms, thereby improving the estimation of elasticity for the tumor region.

### 4.3 Adversarial training

GANs are trained in an iterative adversarial fashion to allow the generator to produce high-quality synthetic samples. In the initialization phase, the generator produces synthetic samples with a distribution similar to the training data using random noise or B-mode US images. In the first step, the discriminator is trained on real and V-EUS samples with the goal of learning to differentiate the two classes accurately. In the second step, the generator is trained to create realistic synthetic samples which the discriminator can classify as real. The adversarial training process allows the generator to perform implicit feature learning, enabling it to detect complex patterns and structures. In the context of EUS generation, the generator does not have information regarding the tissue elasticity explicitly available in the US images; rather, it implicitly learns the complex patterns and correlations between the US images and the desired V-EUS.

### 4.4 Loss function

In the methodology details outlined by Yao et al. (20), the discriminator loss function is the average of tumor and global cross-entropy losses for accurately classifying real or V-EUS. The generator loss function is formulated to maximize the probability of the discriminator classifying generated samples as real. Furthermore, color loss (i.e., L1 loss) between the VEUS and ground truth weighed by color rarity coefficient is added to generator loss for accurate color distribution of the elastograms.

### 4.5 Evaluation metrics

Yao et al. (20) perform a thorough quantitative analysis of V-EUS to validate the GAN framework. Particularly the Structural Similarity Index (SSIM), Mean Absolute Percentage Error (MAPE), and Contrast-to-Histogram Correlation (CHC) are used for quantifying the difference between V-EUS and real EUS. An elaborate explanation of these metrics is provided in the **Supplementary Materials**.

A comprehensive qualitative analysis is conducted subsequent to the quantitative analysis, employing a blind evaluation with the Tsukuba scoring system. This evaluation involves radiologists with diverse levels of experience, ensuring a thorough and unbiased assessment of the V-EUS relative to real EUS. The qualitative analysis validates that the generated EUS has a matching visual appearance to real EUS and gathers feedback from radiologists regarding their preferences. This is crucial for the success of V-EUS because radiologists should be able to incorporate it into their diagnostic workflows and make accurate diagnoses without additional training. Thus, the positive outcomes of the qualitative analysis add to the clinical credibility of the methodology proposed by Yao et al. (20).

## 5 Analysis of results

This section analyzes whether the results presented by Yao et al. (20) support the claims made by the authors. First, the authors highlight that the proposed GAN framework results in SSIM, MAPE, and CHC scores of 0.903, 0.304, and 0.849, respectively, indicating that numerical metrics show a high overlap in distributions between the real and V-EUS. The preferable SSIM and CHC values are due to the use of the color coefficient and color loss, augmented with the tumor discriminator loss, allowing the GAN to put additional emphasis on the elasticity of the tumor region and overall color distribution. The choice of these quantitative metrics is in line with the literature for the synthesis of CT, MRI, and retinal color fundus images (46). However, SSIM evaluates the V-EUS by comparing local patterns of pixel intensities and does not account for global variations in quality. Similarly, MAPE may lead to misinterpretation of errors because the absolute percentage difference does not provide insights regarding the overestimation or underestimation of elasticity. The quantitative analysis would be more meaningful if Yao et al. (20) incorporated metrics such as multi-scale SSIM, which compares both local and global aspects of the image. Furthermore, Yao et al. (20) omit Frechet Inception Distance (FID) from their quantitative analysis, which is a key metric to evaluate the quality of the GAN-generated images as shown by Zhang et al. (38) for elastogram synthesis of the Thyroid. Nevertheless, the results show that the strain ratio (SR) computed from real and V-EUS leads to statistically similar AUC for diagnosing breast tumors, suggesting that V-EUS can replace real EUS in diagnostic scenarios. Additional stratified analysis of breast cancer diagnosis for tumors of varying sizes and at different locations results in similar performance between real and V-EUS, suggesting that V-EUS can overcome the human subjectivity in capturing the EUS by eliminating the variables such as probe position, applied pressure, and frequency of mechanical compression. The stratified analysis also successfully conveys to the readers that the GAN framework generalizes across tumor sizes and locations, which is critical for real-world deployment.

Second, the results validate the GAN’s generalizability with 1730 breast cancer cases across fourteen other medical centers with varying imaging and clinical settings, showing that the GAN framework is independent of perturbations in imaging and clinical settings. Particularly, the authors evaluated the SSIM, MAPE, CHC, and diagnostic AUC for each of the fourteen centers and compared them with the inter-validation performance to assess model generalizability. This thorough analysis of the GAN framework across different medical centers is unique to the study conducted by Yao et al. (20) and is missing from other studies for elastogram synthesis (38). However, the validation sets are completely based on the Chinese population, requiring further validation for other ethnic groups. In line with these results, the authors also show that the GAN can generate V-EUS from low-resolution pocket US images. Adding the V-EUS to the pocket US allows radiologists to improve breast cancer diagnosis by up to 5%, indicating that V-EUS improves the clinical utility of pocket US. Altogether, the outcomes indicate that V-EUS can improve the accessibility and diagnostic accuracy of low-resolution pocket US.

Third, the results incorporate human feedback and evaluation to bridge the gap between computational metrics and human perception. Yao et al. (20) are the first in the literature to perform a novel qualitative analysis to support the quantitative results and usage of V-EUS in radiological workflows. This form of exhaustive qualitative analysis is missing from previous studies for elastogram synthesis (38) and medical image modality translation (34, 35, 41, 44). Specifically, the authors perform a blind evaluation test to compare the preference of junior and senior radiologists between real and V-EUS. Involving radiologists with different experiences allows authors to gather insights into their contrastive preferences in diagnostic workflows. For instance, the study showed that junior radiologists preferred V-EUS over the real EUS for breast cancer diagnosis using the BI-RADS score, thereby validating the feasibility of V-EUS in day-to-day usage for radiologists. The authors also show that V-EUS can be generated for deep-seated tumors (i.e., depth greater than 20 mm) without artifacts. In contrast, 25.9% (62 of 239) of real EUS display artifacts due to signal degradation at greater anatomical depths. This is a significant breakthrough as real EUS with elasticity artifacts could not be used in practice for diagnosing diseases in deep-seated tissues and tumors. Subsequently, V-EUS opens the possibility of carcinoma diagnosis in deep body tissues, which are currently diagnosed by high-definition 3D imaging modalities (i.e., CT or MRI).

Exhaustive analysis of the results and methodology also reveals that Yao et al. (20) provide sufficient details for the reproducibility and validity of the work. Notably, the methodology clearly explains and details the different components of the GAN framework, including network hyperparameters, training hyperparameters, loss function, metrics, etc. Additionally, open-source implementation of the GAN framework is available on GitHub for verifying the results. Furthermore, the dataset used for training the networks is available upon request after agreeing to terms and conditions. However, the lack of clear documentation and comments in the code makes it challenging for the users to decipher the details in the training and evaluation of the network. Overall, the manuscript makes the methodology and results transparent to the scientific community.

## 5 Challenges and future directions

This section highlights the unaddressed challenges and gaps in the literature for synthesizing EUS from B-mode US images. Yao et al. (20) evaluate the malignancy of tumors based on SR. The SR is defined as the ratio of average tumor elasticity and a reference region. The generated EUS is decoded by quantizing the image into 256 pseudo-color levels, representing varying elasticity. However, the authors do not justify whether 256 elasticity values are sufficient for representing the underlying elasticity distribution of breast tissues through experiments or evidence from the literature. Furthermore, the SR is computed without providing any specific guidelines for selecting the reference region. These oversights in generating the SR raise concerns about whether the generated V-EUS can effectively model the physical independent information of the underlying breast tissue. Even though the authors show that the effectiveness of SR extracted from V-EUS in diagnosing breast cancer is similar to real EUS, further validation is necessary to clarify whether the other physical properties of the tissue, such as viscoelasticity, anisotropy, homogeneity, or heterogeneity are correctly modeled. Thus, as the first step, we recommend a comprehensive phantom study for the quantitative validation of V-EUS. By gathering feedback from medical experts regarding the biomechanical properties of the V-EUS, the research community can better understand the utility and potential clinical applications of V-EUS.

One critical pitfall of the GANs is class leakage. The groundbreaking work by Salimans et al. (47) demonstrates that GAN-generated images, initially intended to represent a specific class, exhibit the inclusion of properties and attributes from unrelated classes. This blending of features across distinct modes within the training distribution poses a significant concern, as it may lead to the generation of V-EUS images that display interpolations between malignant and benign tumors. The presence of these intermediate or outlier V-EUS images has the potential to misguide radiologists, resulting in erroneous diagnoses and suboptimal outcomes during biopsies. Such outcomes include harm to healthy tissues or the recurrence of carcinoma. To tackle this challenge, we encourage researchers to draw inspiration from techniques developed for feature disentanglement. Notably, prior studies have successfully enforced disentangled learning from noise vectors by incorporating a regularization term that penalizes the network when modifying a single element leads to changes in multiple features within the generated image. Similarly, we propose adopting regularization strategies to penalize the network for the intermixing of attributes originating from different modes of the training distribution in the generated V-EUS.

Accurate quantitative evaluation of GAN-generated medical images represents a significant challenge within image synthesis literature. This challenge arises due to the limitations of conventional metrics, SSIM, which primarily provides a high-level comparison of images based on luminance and contrast. However, pixel-wise metrics like MAPE may assign low values to blurry generated images, failing to adequately capture the visual quality of synthesized V-EUS images. Consequently, researchers like Yao et al. (20) are compelled to undertake comprehensive qualitative studies to assess image fidelity. In a pioneering study, Zhang et al. (48) have shown that the deep features of neural networks can serve as a foundation for developing perceptual metrics. To elaborate, the authors introduce learned perceptual image path similarity (LPIPS), which achieves better agreement with human perception than conventional metrics like SSIM. Given these advancements, we recommend that researchers embrace the state-of-the-art perceptual metrics for conducting quantitative evaluations of GAN methodologies applied in elastogram generation.

Another critical limitation of deep learning methodologies in medical practice is the black-box nature of neural networks. The network explainability information is critical for radiologists to trust the synthesized output, address any biases, and account for significant errors in the elastograms. To ensure the reliability and interpretability of the generated V-EUS, medical practitioners need to understand the underlying components of the B-mode US that contribute to the network’s decision-making process. GANs learn the mapping between the B-mode US and the V-EUS by implicit feature learning through an adversarial training process, elevating the need to understand the mapping between the US and the synthesized EUS. Recent advancements have demonstrated the integration of explainability techniques into neural network architectures for the fusion of MRI and CT scans (49). Building upon this progress, it is feasible to develop explainable GAN frameworks as an extension to the work conducted by Yao et al. (20). The enhanced transparency will enable medical professionals to foster trust and improve the clinical utility of V-EUS.

One of the strengths of the Yao et al. (20) methodology is the inclusion of a dataset that spans multiple medical centers, thus ensuring the evaluation of their GAN across diverse imaging and clinical parameters. However, the population demographic in these hospitals is limited to Chinese patients, thereby restricting the evaluation of the GAN’s performance to this demographic. Consequently, the generalizability of the proposed GAN network to other populations with potentially distinct lesion characteristics, such as those with deeper lesions compared to the Asian demographic, remains unexplored. Yao et al. (20) have conducted validation experiments specifically focusing on tumors located at several depths up to 20 mm. While these findings provide valuable insights into the performance of the GAN framework at varying depths, it is crucial to conduct further evaluations across different racial populations. Such evaluations would shed light on the ability of the GAN to generate V-EUS images of breast lesions with varying spread and depth distributions in populations beyond the Chinese demographic, contributing to a more comprehensive understanding of the GAN’s capabilities and limitations.

## 6 Conclusion

To summarize, we perform a comprehensive critical review of the GAN-based methodology equipped with color loss for the generation of realistic EUS images for breast lesion diagnosis. Specifically, we briefly review the methods in image reconstruction and medical image super-resolution to understand the progress in deep learning, which has led to the GAN-based methodologies for elastogram generation from B-mode US. Moreover, we analyze whether the claims are well-supported by quantitative and qualitative evaluations. Finally, we highlight the unaddressed challenges and the future directions in elastogram synthesis. As a whole, the critical review provides a clear understanding of the current cutting-edge deep learning framework for the V-EUS generation while paving the pathway for the upcoming research in elastography synthesis.

## Author contributions

MA: Conceptualization, Data curation, Formal analysis, Investigation, Methodology, Resources, Validation, Visualization, Writing – original draft, Writing – review & editing. MQ: Conceptualization, Formal analysis, Supervision, Validation, Writing – review & editing. RR: Conceptualization, Formal analysis, Supervision, Writing – review & editing. ES: Conceptualization, Supervision, Writing – review & editing. KQ: Conceptualization, Formal analysis, Funding acquisition, Supervision, Writing – review & editing.
