# Supplementary material for: Unveiling the future of breast cancer assessment: a critical review on generative adversarial networks in elastography ultrasound
Source: Front Oncol. 2023 Dec 6;13:1282536. doi: 10.3389/fonc.2023.1282536 (PMC10731303; doi:10.3389/fonc.2023.1282536)
Supplement: Supplementary file 1 [file Image_1.pdf]

A comprehensive explanation of quantitative metrics is as follows:

SSIM: The metric accounts for the similarity between the luminance and contrast of the real EUS and V-EUS images to quantify the similarity.

$$SSIM(x,y) = \frac{(2\mu_x\mu_y + C_1)(2\sigma_{xy} + C_2)}{(\mu_x^2 + \mu_y^2 + C_1)(\sigma_x^2 + \sigma_y^2 + C_2)}, \quad (1)$$

where the numerator capture the intensity/luminance similarity between images using means (μ) and the contrast information through covariance (σ_{xy}). The denominator scales the metric with respect to average luminance and contrast (σ_x,σ_y) of the two images.

MAPE: This metric captures the average mean absolute error between stain ratios of the real EUS and V-EUS.

$$MAPE = \frac{1}{n}\sum_{i=1}^{n}\left|\frac{y_i - \hat{y}_i}{y_i}\right|, \quad (2)$$

where n denotes the total count in the elastgram. Variables y_i and $\hat{y}_i$ represent the strain scores of the ith location in the real and virtual elastograms, respectively.

CHC: This metric measures the correlation between the histograms of real and virtual EUS in the hue and saturation color space:

$$CHC = 1 - \sqrt{1 - \frac{\sum\sqrt{C_{real}.C_{virtual}}}{\sqrt{\sum C_{real}.\sum C_{virtual}}}}, \quad (3)$$

where C_{real} and $C_{virtual}$ are vectors corresponding to the histogram bin count of real EUS and V-EUS, respectively.
